# Editorial: Molecular markers in rheumatic diseases and their comorbidities

**DOI:** 10.3389/fmed.2023.1266563

**Published:** 2023-09-12

**Authors:** Monica Vazquez-Del Mercado, Erika Aurora Martínez-García

**Affiliations:** ^1^Departamento de Biología Molecular, Centro Universitario de Ciencias de la Salud, Instituto de Investigación en Reumatología y del Sistema Músculo Esquelético, Universidad de Guadalajara, Guadalajara, Jalisco, Mexico; ^2^Hospital Civil de Guadalajara “Dr. Juan I. Menchaca”, Especialidad de Reumatología, Sistema Nacional de Posgrados (SNP) 004086, Consejo Nacional de Humanidades, Ciencias y Tecnologías (CONAHCYT), Guadalajara, Jalisco, Mexico; ^3^Universidad de Guadalajara-Cuerpo Académico (UDG-CA)-703, Inmunología y Reumatología, Centro Universitario de Ciencias de la Salud, Universidad de Guadalajara, Guadalajara, Jalisco, Mexico; ^4^Departamento de Fisiología, Centro Universitario de Ciencias de la Salud, Universidad de Guadalajara, Guadalajara, Jalisco, Mexico

**Keywords:** molecular markers, rheumatic diseases, comorbidities, inflammation, autoimmunity

Biomarker research in the field of rheumatic diseases is an emerging topic due to its relevance in the evaluation of preclinical stages, clinical outcomes, and prognosis (1). In this regard, the Research Topic “Molecular Markers in Rheumatic Diseases and Their Comorbidities” presents a collection of interesting articles.

Systemic autoimmune rheumatic diseases (SARD) are a group of pathologies characterized by an inflammatory condition that involves overactivated or suppressed genes, DNA functional changes, HLA class I and II associations, and a bulk of autoantibodies against nuclear and cytoplasmic components (2, 3). It is well-known that other chronic inflammatory conditions may trigger or enhance the inflammatory state of SARD. Examples of some of these comorbidities are vascular aging, increased arterial stiffness, metabolic syndrome, etc. (4–8). This constitutes a real challenge to clinicians because, in addition to managing SARD, they have to offer adequate control of comorbidities in a multidisciplinary setting with the aim of improving the quality of life of their patients (9). Liang et al. showed elevated levels of human epididymis protein 4 (HE_4_), a protein expressed in several tissues and used as a biomarker in ovarian cancer (10), in patients with rheumatoid arthritis (RA) compared to healthy subjects; those who were positive for HE_4_ had a higher prevalence of interstitial lung disease (ILD); these data are the first to identify a correlation between HE_4_ levels and ILD.

In recent years, vitamin D deficiency has been associated with pleiotropic dysfunction or health issues such as suicidal ideation, bone turnover, etc. (11). In this Research Topic, Diao et al. carried out a systematic review and meta-analysis of vitamin D deficiency between ankylosing spondylitis (AS) patients and healthy controls, proposing a possible protective role of vitamin D in AS.

The achievement of remission is one of the clinical outcomes of the management of patients with rheumatic diseases. Su et al. studied a possible biomarker for clinical remission in adult-onset Still's disease (AOSD) through the measurement of cysteine-rich angiogenic inducer 61 (Cyr61) levels (12). They identified elevated levels of Cyr61 in asymptomatic AOSD patients and showed an inverse correlation of Cyr61 with proinflammatory IL-1β, IL-6, and IL-17. Therefore, the authors highlighted the involvement of Cyr61 in tissue repair.

Some biomarkers can be detected in serum 9–10 years before the clinical onset of the disease, for example as antinuclear antibodies (13) or anti-citrullinated peptide antibodies (14). Another example discussed in this Research Topic is an inhibitor of apoptosis: survivin. Erlandsson et al. suggested that survivin shows a strong clinical association with arthralgias in preclinical RA.

MicroRNAs (miRNAs) have been associated with a myriad of inflammatory rheumatic diseases and their comorbidities, which include cardiovascular diseases (15). In the extensive research by Li et al., 42 up-regulated and 45 down-regulated miRNAs were found in AS compared with controls. Finally, García-Ortiz et al. studied the Xq28 risk haplotype in the Mexican population associated with susceptibility to childhood-onset systemic lupus erythematosus.

## Author contributions

MV-M: Writing—original draft, Writing—review and editing. EM-G: Writing—original draft, Writing—review and editing.
